# IADL DIFFICULTY AND MORTALITY IN ACTIVE: IMPLICATIONS FOR TRANSFER OF COGNITIVE TRAINING

**DOI:** 10.1093/geroni/igz038.1615

**Published:** 2019-11-08

**Authors:** Alden L Gross, Olivio Clay, Cynthia Felix, Fred Unverzagt, Michael Marsiske, Norma Coe

**Affiliations:** 1 Johns Hopkins Bloomberg School of Public Health, Baltimore, Maryland, United States; 2 University of Alabama, Birmingham, Alabama, United States; 3 Indiana University, Indianapolis, Indiana, United States; 4 University of Florida, Gainesville, Florida, United States; 5 University of Pennsylvania, Pennsylvania, Pennsylvania, United States

## Abstract

Objectives: Our goals were to externally scale an IADL difficulty scale to the more recognizable Functional Activities Questionnaire (FAQ), and test whether cognitive training attenuates the relationship between IADL difficulty and mortality. Method: We leveraged externally available FAQ data from NACC to scale questions about IADL activities administered in ACTIVE (N=2,802) using item response theory. We modeled time to death as a function of IADL difficulty in ACTIVE using survival analysis, testing whether ACTIVE intervention group status modified the association between FAQ and mortality. Results: IADL difficulty in ACTIVE, scaled to the FAQ, was associated with a higher risk of death (Hazard Ratio, HR, 1.01, 95% Confidence Interval, CI: 1.001, 1.02). The relationship did not differ by ACTIVE intervention status. Discussion: Cognitive training does not modify the relationship between IADL difficulty and mortality, consistent with a hypothesis that proposed relationships between cognitive ability and IADL difficulty are correlational, not causal.

